# Safety and efficacy of transaxillary transcatheter aortic valve replacement using a current-generation balloon-expandable valve

**DOI:** 10.1186/s13019-020-01291-z

**Published:** 2020-09-10

**Authors:** Yong Zhan, Nicholas Toomey, Jamel Ortoleva, Masashi Kawabori, Andrew Weintraub, Frederick Y. Chen

**Affiliations:** 1 Division of Cardiac Surgery, CardioVascular Center, Tufts Medical Center, Tufts University School of Medicine, 800 Washington Street, #266, Boston, MA 02111 USA; 2 Division of Cardiac Anesthesia, Tufts Medical Center, Tufts University School of Medicine, Boston, MA USA; 3 Division of Cardiology, CardioVascular Center, Tufts Medical Center, Tufts University School of Medicine, Boston, MA USA

**Keywords:** Transcatheter aortic valve replacement, Transaxillary, Alternative access, Balloon-expandable valve, SAPIEN 3

## Abstract

**Background:**

Transaxillary access (TAx) has shown promise as an excellent alternative TAVR option, but data on the Edwards SAPIEN 3 in TAx-TAVR is limited. We sought to study the safety and efficacy of TAx-TAVR using this current-generation balloon-expandable valve.

**Methods:**

A retrospective study of our first 24 TAx and 20 transthoracic (TT) TAVR patients treated with the SAPIEN 3 valve was performed, and the patients’ preoperative characteristics, procedural outcomes, and clinical outcomes were compared to our first 100 transfemoral (TF) patients using the SAPIEN 3 device.

**Results:**

There were no statistical differences observed for outcomes between the TAx and TF groups, despite the TAx patients having more comorbidities (STS-PROM 11.3 ± 7.6 versus 7.3 ± 5.2, *p* = 0.042). In addition, no significant difference was found in the fluoroscopy time and contrast amount between the two groups. The patients’ baseline characteristics were similar between the TAx and TT groups. Their procedural and clinical outcomes were comparable, but there was a trend towards lower incidence of acute kidney injury (13.0% versus 23.5%), new-onset atrial fibrillation (5.6% versus 33.3%), shorter median length of stay postoperatively (4 versus 6 days), fewer discharges to rehabilitation (16.7% versus 35.0%), and a lower rate of readmission within 30-days (8.3% versus 35.0%), all favoring TAx access.

**Conclusions:**

TAx-TAVR with the SAPIEN 3 valve is a safe alternative to TF access. It offers advantages of improved recovery over TT access, and appears to be a superior alternative-access option for TAVR. TAx access could be preferred when TF access is not feasible.

## Introduction

Transcatheter aortic valve replacement (TAVR) has become a major therapy for patients with severe aortic valve stenosis, and the indications continue to evolve [1]. A variety of approaches must be considered when performing a TAVR procedure, including the method of vascular access as well as the type of device. While transfemoral access (TF) remains the default delivery route for TAVR, it is not always feasible. In such circumstances, alternative TAVR routes including transapical (TA), transaortic (TAo), and transaxillary (TAx) approaches have been utilized [2, 3]; however, there is no consensus on the selection criteria for these approaches to TAVR.

TAx access is preferred over other non-transfemoral approaches in the setting of TAVR using self-expandable transcatheter valves [4]. There is growing evidence on the safety and efficacy of TAx-TAVR using balloon-expandable valves, but an individual operator’s experience with the current-generation SAPIEN 3 valve (Edwards Lifesciences, Irvine, CA, USA) in TAx-TAVR is still limited [5]. In addition, much of the experience in TAx-TAVR was derived from studies focusing on left TAx access, which provides more coaxial orientation for device insertion, and therefore, is the predominant TAx approach [6, 7]. We have developed a technique for right TAx-TAVR using the SAPIEN 3 valve to overcome the vascular tortuosity and unfavorable implantation angle inherent to this approach, and thus have expanded the patient population for the TAx approach [8].

While preferences for alternative TAVR approaches vary from institution to institution, tailoring TAVR to anatomic considerations and carefully choosing alternative access routes is critical to reduce vascular complications and facilitate recovery. As TAVR outcomes can be device and access-specific [9], we studied our institutional experience with the use of the SAPIEN 3 valve in various TAVR approaches, and sought to determine whether our current algorithm of alternative-access selection was effective.

## Materials and methods

### Patient selection

This is a single-center, retrospective, and comparative study of patients treated consecutively with the SAPIEN 3 TAVR via alternative-access routes. In a total of 474 TAVR patients who underwent TAVR procedures between August 2015 and June 2019 at our institution, 24 had TAx-TAVR and 20 had transthoracic (TT-) TAVR, including TA and TAo approaches. They were compared to our first 100 TF patients undergoing SAPIEN 3 TAVR during the study period.

**Fig. 1:**
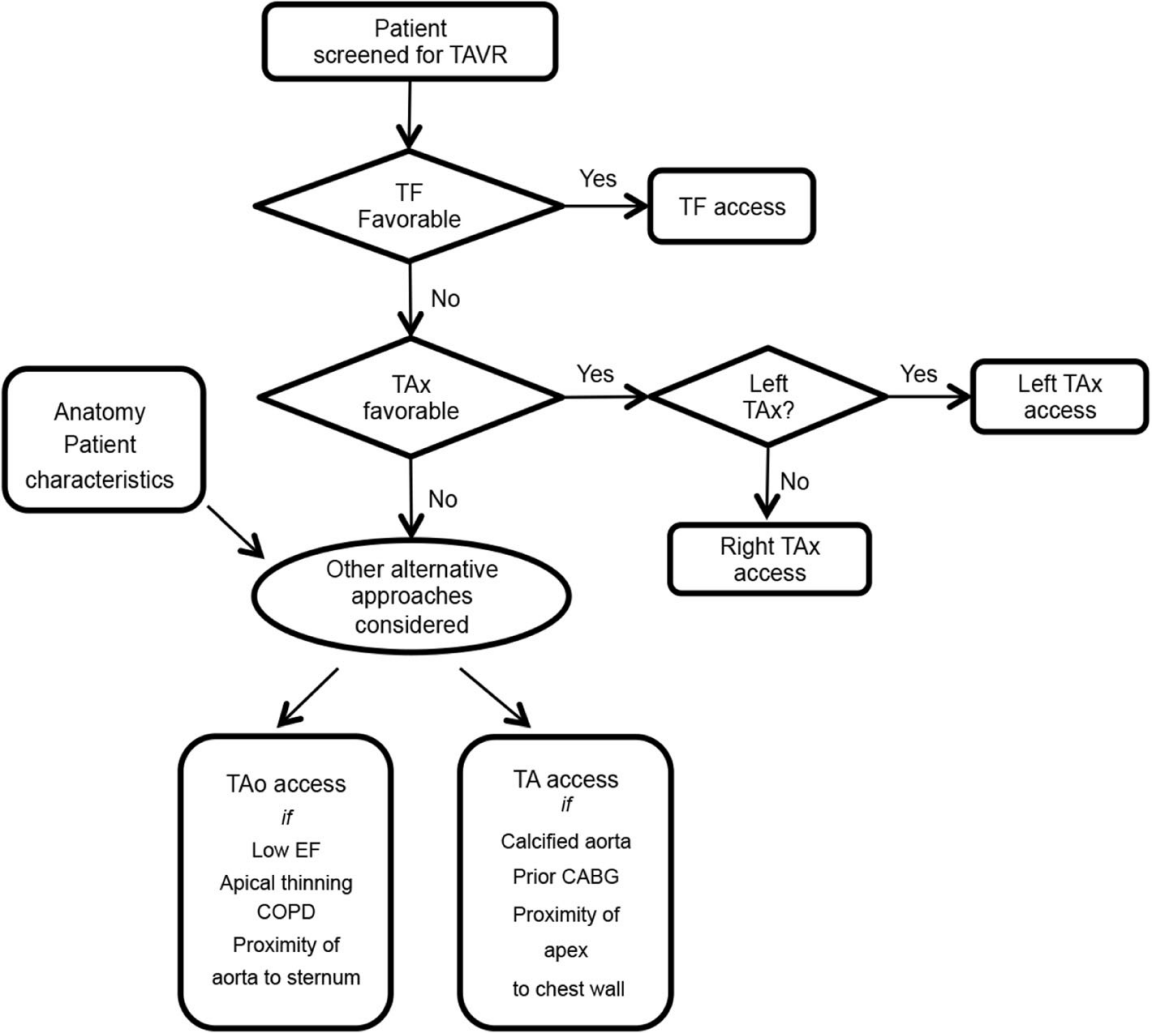
Flowchart displays the algorithm of alternative access selection at our institution. TAVR, transcatheter aortic valve replacement; TF, transfemoral; TA, transapical; TAo, transaortic; TAx, transaxillary; EF, ejection fraction; COPD, chronic obstructive pulmonary disease

The selection of an access route was at the discretion of our multi-disciplinary team, according to patient characteristic and anatomical considerations (Fig. 1). Patients who were not amenable to TF-TAVR were screened for TAx access, which was considered over TT access (TAo or TA) at our institution. Left TAx access was favored, and right TAx access was chosen when the left axillary artery was < 5.5 mm or < 6.5 mm when there was a patent LIMA-dependent flow to the coronary artery. Vascular tortuosity was less of a concern, as it could be generally straightened by the rigid wire; however, calcification and small caliber could preclude TAx access. Selection of TAo versus TA access was determined based on patient characteristics and preoperative imaging data. TAo access was favored in patients with poor ventricular functions, underlying pulmonary issues, thinning or scarring of the apex of the heart, and close proximity of the aorta to the sternum, whereas TA access was preferred if the aorta was calcified or had patent bypass grafts, or the apex of the heart was adjacent to the intercostal space.

### Surgical technique

**Fig. 2:**
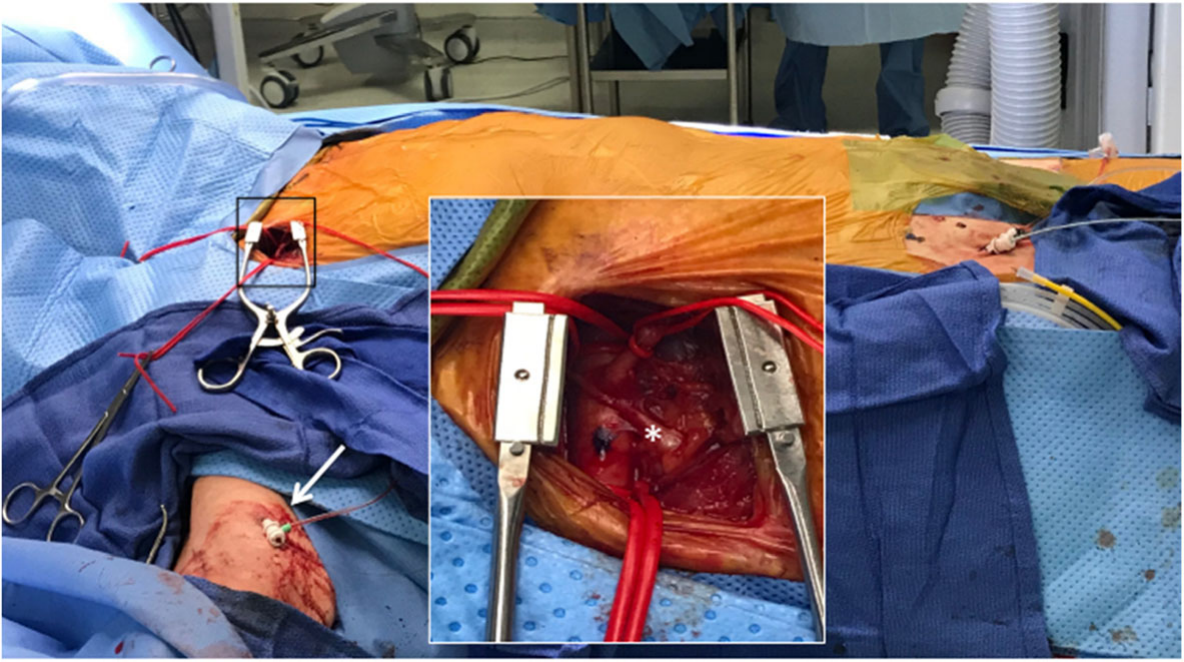
Operative approach to transaxillary TAVR. The axillary artery is exposed via surgical cut-down, and the anatomy is shown in the inset. Note the brachial nerve (*) is in the vicinity of the axillary artery. Access to the brachial artery (arrow) is obtained in selected cases due to concerns for vascular complications

The procedural technique for TAx-TAVR has been reported previously [10]. In brief, it is performed under general anesthesia via a surgical cut-down to expose the axillary artery for direct puncture (Fig. 2). When there are anatomical concerns, such as tortuosity, small caliber, or calcification of the subclavian artery, we place a V-18™ wire (Boston Scientific, Marlborough, MA, USA) via the ipsilateral brachial artery to the abdominal aorta to guide endovascular interventions as necessary (Fig. 2, arrow). We used the ‘flip-n-flex’ technique in right TAx-TAVR to facilitate coaxial alignment of the prosthesis and aortic annulus [8]. Primary repair of the axillary artery followed by angiography to confirm a successful repair was performed at the completion of the procedure.

Patients who were ineligible for the TAx approach were evaluated for either the TA or TAo approach. The TAo approach was performed via a small right thoracotomy in the 2nd intercostal space or a short J-shaped manubriotomy, based on the anatomical relationship of the ascending aorta to the sternum. The TA approach was performed via a thoracotomy through the intercostal space corresponding to the apex of the heart. The SAPIEN 3 device was inserted through the Certitude sheath (Edwards LifeSciences, CA, USA). The technical aspects of the TA and TAo approaches for balloon-expandable valves have been previously described in the literature [11].

TF-TAVR was performed via percutaneous access, and closure of the femoral artery was performed using the ProGlide® Perclose device (Abbott Vascular, Santa Clara, CA, USA).

### Outcomes

Study outcomes were 30-day mortality and complication rates, according to the Valve Academic Research Consortium (VARC)-2 definitions [12]. Fluoroscopy time, amount of contrast use, procedural time, postoperative length of stay, discharges to rehabilitation facilities, and readmissions were also studied.

### Statistical analysis

Categorical data are presented as numbers and percentages, and continuous data as mean ± standard deviation (SD) or as median and interquartile range (IQR). Comparisons among three groups were performed using Pearson χ^2^ test and Student’s t test or Kruskal-Wallis H test (non-parametric data). For comparisons between two groups, Pearson χ^2^ test or Fisher’s exact test and Student’s t test or Mann-Whitney U test (non-parametric data) were used, whenever appropriate. Results were considered as statistically significant when the *p*-value was less than 0.05. Data analyses were performed using SPSS statistics software version 24 (IBM Corporation, Armonk, NY, USA).

## Results

### Study cohort

Among the 24 patients in the TAx group, 10 were treated with left TAx-TAVR and 14 right TAx-TAVR procedures. The TT group included 15 TA and 5 TAo patients. Given the small numbers, the TA and TAo patients were grouped as one cohort for analysis. Literature suggests that these two TAVR approaches do not differ in major procedural and clinical outcomes [13, 14].

### Baseline characteristics

The baseline characteristics of the three groups are summarized in Table 1. The STS-PROM scores of the TAx and TT groups were statistically higher than the TF group (11.3 ± 7.6 and 11.9 ± 11.8 versus 7.3 ± 5.2, *p* = 0.042 and *p* = 0.026, respectively). Compared to the TF group, the TAx group had higher rates of diabetes (62.5% versus 38%, *p* = 0.029), chronic kidney disease (45.8% versus 23%, *p* = 0.024), chronic lung disease (58.3% versus 23%, *p* = 0.001), and history of percutaneous coronary intervention (45.8% versus 19%, *p* = 0.006). The TT group was significant for the prevalence of end-stage renal disease (15% versus 2% for TF, *p* = 0.032). The baseline characteristics between the TAx and TT groups were comparable, except for more patients with diabetes in the TAx group (*p* = 0.012). There were no significant differences among the preoperative echocardiographic data of the three groups. The TAx group had higher percentage of patients with pulmonary artery systolic pressure (PASP) > 40 mmHg, compared to the TF group (45.8% versus 14%, *p* = 0.001).

**Table 1:** Patient Demographic and Preoperative Characteristics

Variable	TF (*n* = 100)	TAx (*n* = 24)	TT (*n* = 20)	*p* Value	p Value between groups
Age, years	80.5 ± 7.6	82.9 ± 8.8	81.3 ± 5.3	0.386	
Male	48 (48)	10 (41.7)	9 (45)	0.846	
Body mass index, kg/m^2^	28.8 ± 6.2	27.1 ± 6.2	25.8 ± 5.0	0.095	
Body surface area, m^2^	1.9 ± 0.2	1.8 ± 0.2	1.8 ± 0.3	0.208	
Hypertension	86 (86)	20 (83.3)	20 (100)	0.179	
Diabetes	38 (38)	15 (62.5)	5 (25)	0.029	TF vs TAx, 0.029; TAx vs TT, 0.012
Coronary artery disease	76 (76)	19 (79.2)	16 (80)	0.895	
Chronic kidney disease	23 (23)	11 (45.8)	9 (45)	0.025	TF vs TAx, 0.024; TF vs TT, 0.042
End-stage renal disease	2 (2)	1 (4.2)	3 (15)	0.029	TF vs TT, 0.032
Creatinine^a^, mg/dL	1.3 ± 1.1	1.6 ± 0.9	1.6 ± 1.6	0.291	
Chronic lung disease	23 (23)	14 (58.3)	8 (40)	0.002	TF vs TAx, 0.001
Cerebrovascular accident	11 (11)	5 (20.8)	5 (25)	0.171	
Previous cardiac surgery	17 (17)	5 (20.8)	7 (35)	0.186	
Previous CABG	16 (16)	4 (16.7)	7 (35)	0.133	
Previous PCI	19 (19)	11 (45.8)	7 (35)	0.015	TF vs TAx, 0.006
Permanent pacemaker	11 (11)	0 (0)	0 (0)	0.073	
Atrial fibrillation	35 (35)	6 (25.0)	5 (25)	0.495	
NYHA III/IV	90 (90)	22 (91.7)	18 (90)	0.969	
STS-PROM	7.3 ± 5.2	11.3 ± 7.6	11.9 ± 11.8	0.005	TF vs TAx, 0.042; TF vs TT, 0.026
**Echocardiographic data**					
Aortic valve area, cm^2^	0.7 ± 0.2	0.8 ± 0.1	0.7 ± 0.2	0.354	
Peak gradient, mmHg	68.9 ± 23.4	60.6 ± 19.4	66.7 ± 20.4	0.259	
Mean gradient, mmHg	39.2 ± 13.9	35.5 ± 11.6	39.7 ± 14.0	0.378	
Peak velocity, m/s	4.1 ± 0.7	3.8 ± 0.6	4.0 ± 0.7	0.229	
LVEF, %	54.8 ± 11.6	53.0 ± 11.1	49.0 ± 15.4	0.142	
PASP> 40 mmHg	14 (14)	11 (45.8)	6 (30)	0.002	TF vs TAx, 0.001

^a^ Creatinine level is the most recent value before surgery

Values are mean ± SD or n (%)

*CABG* coronary artery bypass graft, *PCI* percutaneous coronary intervention, *LVEF* left ventricular ejection fraction, *NYHA* New York Heart Association classification, *PASP* pulmonary artery systolic pressure, *STS-PROM* Society of Thoracic Surgeons predicted risk of mortality score, *TAx* transaxillary, *TF* transfemoral, *TT* transthoracic

### Procedural outcomes

The major procedural outcomes are summarized in Table 2. Device success rates were similar among the TF, TAx, and TT groups (96% versus 95.8% versus 95%, *p* = 0.683). No significant differences were observed among the three groups with regard to bleeding events, vascular complications, cerebrovascular accidents, or permanent pacemaker implantation rates. Major vascular complications were rare; 2 TF patients had femoral artery stenosis due to the closure device, 1 TAx patient had focal dissection of the axillary artery required patch repair, and 1 TAo patient had femoral artery thrombosis. None of the patients in all three groups had coronary occlusion, whereas 4% of patients in the TF group and 1 out of 24 (4.2%) in the TAx group underwent planned concurrent percutaneous coronary interventions. All patients had trace or mild paravalvular leak at the end of the procedure and follow-up at 30 days. There were no differences found among the TF, TAx, and TT groups in postprocedural transvalvular mean gradients (10.2 ± 4.4 versus 8.7 ± 3.2 versus 9.2 ± 3.6 mmHg, *p* = 0.187).

**Table 2:** Procedural and Clinical Outcomes

Variable	TF (n = 100)	TAx (n = 24)	TT (n = 20)	p Value
VARC-2 outcomes				
Device success	98 (98)	23 (95.8)	19 (95)	0.683
Bleeding	5 (5)	1 (4.2)	0 (0)	0.593
Life-threatening bleeding	0 (0)	0 (0)	0 (0)	…
Major bleeding	3 (3)	0 (0)	0 (0)	0.510
Minor bleeding	2 (2)	1 (4.2)	0 (0)	0.625
Vascular complication	6 (6)	1 (4.2)	1 (5)	0.933
Major	3 (3)	1 (4.2)	1 (5)	0.887
Minor	3 (3)	0 (0)	0 (0)	0.510
Cerebrovascular accident	2 (2)	0 (0)	1 (5)	0.510
Coronary artery obstruction	0 (0)	0 (0)	0 (0)	…
Pacemaker insertion^a^	8/89 (8.9)	4 (16.7)	5 (25)	0.125
Paravalvular leak (<moderate)	100 (100)	24 (100)	20 (100)	1.000
Acute kidney injury^b^	5/98 (5.1)	3/23 (13.0)	4/17 (23.5)	0.032
Stage 1	3 (3)	2 (8.3)	3 (15)	0.048
Stage 2	2 (2)	1 (4.2)	1 (5)	0.617
30-day Mortality	2 (2)	0 (0)	1 (5)	0.510
Other outcomes				
Transfusion	5 (5)	0 (0)	2 (10)	0.305
New-onset atrial fibrillation^c^	3/65 (4.6)	1/18 (5.6)	5/15 (33.3)	0.002
Postoperative NYHA III/IV	9 (9)	2 (8.3)	2 (10)	0.982
Mean gradient, mmHg	10.2 ± 4.4	8.7 ± 3.2	9.2 ± 3.6	0.187
Discharge to rehabilitation	14 (14)	4 (16.7)	7 (35)	0.077
30-day readmission	8 (8)	2 (8.3)	7 (35)	0.002

Patients with permanent pacemaker^a^, end-stage renal disease^b^, or atrial fibrillation^c^ at baseline are excluded

Values are mean ± SD or n (%)

*NYHA* New York Heart Association classification, *PPM* permanent pacemaker, *TAx* transaxillary, *TF* transfemoral, *TT* transthoracic, *VARC* Valve Academic Research Consortium

As shown in Table 3, fluoroscopy time was shorter with the TT group (12.4 ± 5.0 min) compared to the TF and TAx groups (21.0 ± 5.4 min and 23.9 ± 9.3 min, *p* < 0.001). Similarly, contrast amount was significantly less in the TT group (126.0 ± 45.4 ml) than the TF and TAx groups (162.6 ± 62.5 ml and 155.4 ± 44.9 ml, *p* = 0.039). The overall procedural duration was shorter with the TF group (112.0 ± 28.3 min vs. 185.3 ± 36.9 min for TAx and 156.5 ± 35.6 min for TT; p < 0.001 and *p* = 0.004, respectively).

**Table 3:** Comparison of TAVR Approaches on Procedural and Clinical Efficiency

Variables	TF (n = 100)	TAx (n = 24)	TT (n = 20)	p value	TF vs. TAx	TAx vs. TT	TF vs. TT
Fluoroscopy time, min	21.0 ± 5.4	23.9 ± 9.3	12.4 ± 5.0	< 0.001	0.105	< 0.001	< 0.001
Contrast amount, mL	162.6 ± 62.5	155.4 ± 44.9	126.0 ± 45.4	0.039	0.850	0.217	0.029
Procedural time, min	112.0 ± 28.3	185.3 ± 36.9	156.5 ± 35.6	< 0.001	< 0.001	0.047	0.004
Post-procedural LOS, d	3 (2–4)	4 (3–6)	6 (4–7)	< 0.001	< 0.001	0.132	< 0.001

Values are mean ± SD or median (interquartile range)

*LOS* length of stay, *TAx* transaxillary, *TF* transfemoral, *TT* transthoracic

### Clinical outcomes

**Fig. 3:**
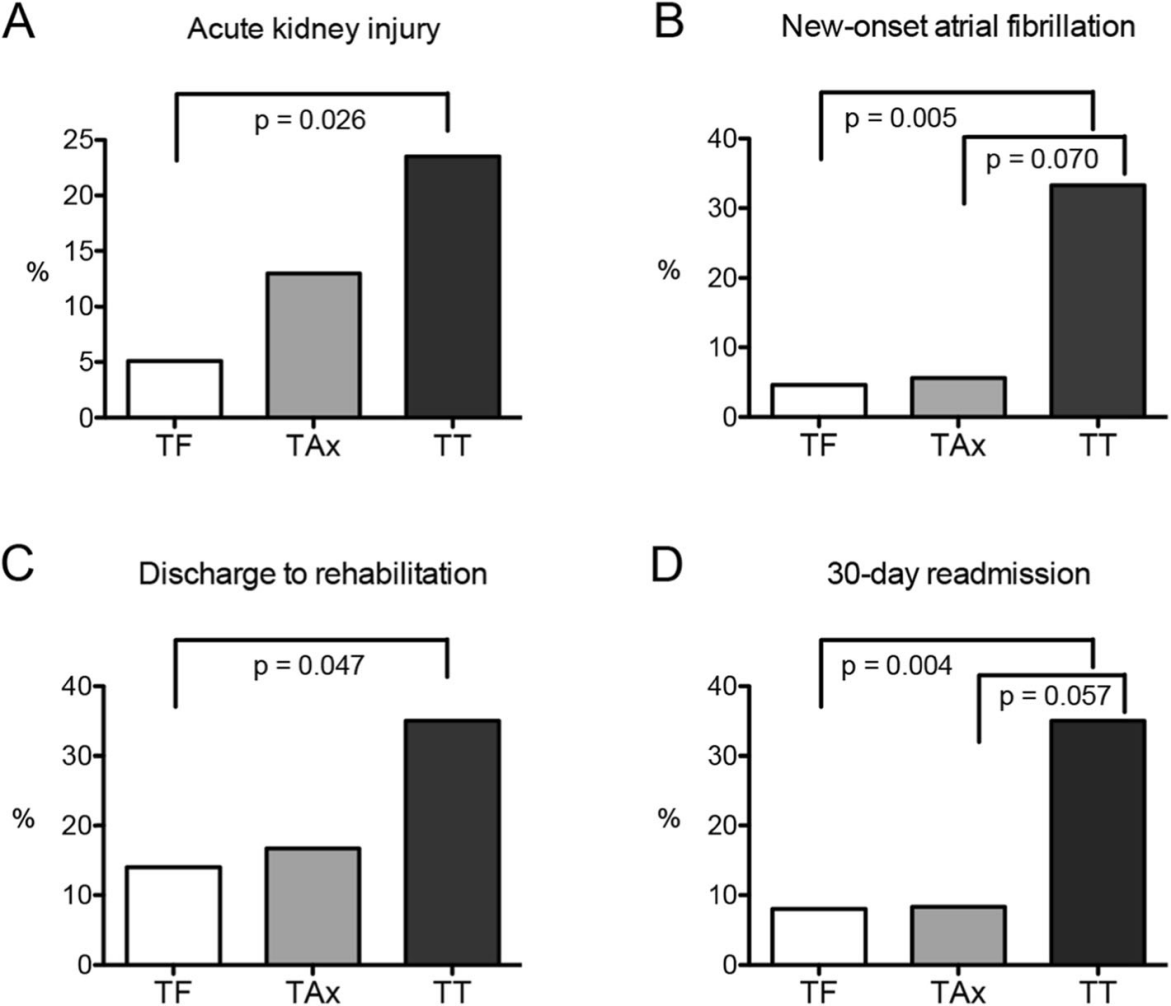
Comparison of postoperative outcomes between TAVR approaches. **a** Acute kidney injury. **b** New-onset atrial fibrillation. **c** Discharge to rehabilitation. **d** 30-day readmission. TF, transfemoral; TAx, transaxillary; TT, transthoracic

Clinical outcomes of the three groups are shown in Table 2. Statistical analyses were also performed between two groups (Fig. 3). The 30-day mortality rates of the three groups were not different (1% versus 0% versus 5%, TF versus TAx versus TT, *p* = 0.510). Less than 10% of the patients in each group continued to have New York Heart Association (NYHA) class III or IV symptoms (*p* = 0.982).

The occurrences of new-onset atrial fibrillation (5.6% versus 4.6%, *p* = 0.869), discharge to rehabilitation facility (16.7% versus 14%, *p* = 0.750), and 30-day readmission (8.3% versus 8%, *p* = 0.957) were comparable between the TF and TAx groups. The TAx group had numerically, but not statistically, higher rate of acute kidney injury (AKI) in comparison to the TF group (13.0% versus 5.1%, *p* = 0.175). Between the TAx and TT groups, there was a trend towards a lower incidence of new-onset atrial fibrillation, fewer discharges to rehabilitation facilities, and a lower readmission rate within 30-days, favoring the TAx group, although the differences did not reach statistical significance (Fig. 3). Compared to the TF group, the TT group was associated with a higher incidence of AKI (23.5% versus 5.1%, *p* = 0.026), new-onset atrial fibrillation (33.3% versus 4.6%, *p* = 0.005), discharge to rehabilitation facility (35% vs. 14%, *p* = 0.047), and 30-day readmission (35% versus 8%, *p* = 0.004) (Fig. 3).

The TAx and TT groups both had longer postprocedural hospital length of stay (LOS) than the TF group (*p* < 0.001). Although non-significant, the TAx group had a shorter median LOS than the TT group (4 versus 6 days, *p* = 0.132).

## Discussion

TAVR devices have improved dramatically over time, which allows the vast majority of device deliveries through femoral arteries. However, there are still patients with peripheral vascular disease who are not candidates for TF-TAVR. In our study period of the SAPIEN 3 platform, alternative-access TAVRs comprised approximately 10% of our total TAVR volume. With the results of recent clinical trials leading to the approval of TAVR in patients at lower surgical risks, the indications for TAVR continue to expand [15, 16]. This further lowered the risk-profile of patient cohorts, in particular, the patients undergoing TF-TAVR. Therefore, we selected our first 100 TF-TAVR patients for a better comparison with the alternative-access patients who tended to have higher STS-PROM scores. In addition, this study represents our initial experience with the SAPIEN 3 valve in the individual access route available at our institution.

While the left TAx approach with the SAPIEN 3 valve has become our alternative-access of choice, certain patients have anatomical or pathological features of the left subclavian artery that make this approach undesired. In such circumstances, the right axillary artery is considered as the next step in our practice. The overall outcomes of our TAx and TF cohorts were comparable, despite that higher incidences of comorbidities were seen with the TAx cohort. This supports the notion that the TAx approach continues to be a preferred alternative-access option in the current era of newer-generation devices. Previous studies comparing the procedural and clinical outcomes of the TAx and TF approaches demonstrated that TAx-TAVR is associated with overall similar outcomes to TF-TAVR using self-expandable valves [4].

Patients who underwent alternative-access TAVR were associated with higher risk-profile. However, equivalent technical success could be achieved with all approaches. In comparison to the TF group, the TT but not TAx group had a significantly higher incidence of new-onset atrial fibrillation. Our result not only is in agreement with the finding of a previous report in which the TA and TAo approaches are associated with increased risk for new-onset atrial fibrillation [5], but also serves as evidence that TAx-TAVR does not differ from TF-TAVR in regard to major outcomes. The higher risk-profiles did not necessarily translate into higher postoperative complications for the TAx patients. We observed that more patients in the TAx and TT groups had AKI after the TAVR procedure. This finding did not correlate with contrast amount or duration of the procedure. Reduction in contrast amount could be off-set by the higher prevalence of chronic kidney disease in the two groups at baseline, which may rationalize the AKI rate of the TAx group, but does not appear to account for that of the TT group. In the literature, higher incidences of AKI have been reported for TA- or TAo-TAVR than TAx-TAVR [17, 18].

The alternative-access of choice for TAVR remains to be elucidated [2, 3]. We achieved comparable procedural outcomes with different TAVR approaches, but the patients who underwent the TT approach required more complex patient care postoperatively, as evidenced by longer LOS and higher rates of readmission and discharge to rehabilitation facility (Fig. 3). Studies revealed that TA- or TAo-TAVR could be associated with higher mortality and decreased survival [5, 17, 19]. Our liberal use of the left and right TAx approaches allowed us to avoid thoracic access in the majority of cases (24/44, 54.5%) where alternative-access routes were deemed necessary. However, when the right TAx approach is also precluded, selection of next available alternative access becomes critical. Transcaval and transcarotid access routes have been explored and shown promising results [20, 21]. More evidence suggests that patients that underwent these peripheral TAVR approaches can benefit from the reduced invasiveness, although procedural and clinical effectiveness require further evaluation [22]. A study comparing the transcarotid and TAx approaches revealed comparable outcomes [23]. Data on the efficacy and safety of either of these approaches is scant, and there were no randomized control studies comparing alternative TAVR approaches to date. Choosing an alternative TAVR route is based on its availability at an individual institution, and depends on a surgical team’s proficiency in performing the procedure.

The current study cohort represents our initial experience with the TAx approach, whereas TA and TAo had been performed with previous-generation balloon-expandable valves. Low-profile delivery systems facilitate the conduct of the procedure. An improvement in the fluoroscopy time in right TAx-TAVR owing to the ‘flip-n-flex’ technique was described in our previous report [10]. As a result, the fluoroscopy time of the entire TAx cohort was similar to the TF cohort. On the other hand, the shorter procedural time of the TT cohort relates to the relatively straightforward insertion of the delivery system and our pre-existing experience with the TA and TAo approaches. Each approach has unique features, and technical challenges could be anticipated in individual access. Familiarity with axillary access facilitates our adoption of the TAx approach, as the axillary artery is used routinely as an alternative cannulation site. We currently perform TAx-TAVR via surgical cut-down. It has been reported that TAx-TAVR can be performed percutaneously, albeit covered stent placement is required in more than 10% of cases [24]. In contrast, the open technique carries a lower risk of bleeding or vascular complications, but potentially at the cost of longer recovery.

Vascular complications remain a concern in the current era despite continuous improvement in the delivery devices. This calls for a broader use of alternative-access and strengthens the notion that TAVR access should be carefully selected to ensure procedural safety. Our experience demonstrates that TAx access is safe and effective using the current-generation TAVR device. A judicious selection of the laterality for TAx-TAVR potentially reduces adverse vascular events and expands the use of this less-invasive TAVR approach. With accumulated experience, alternative TAVR approaches such as TA or TAo can also be safely and effectively performed. However, the TAx approach offers advantages over the TT approach with improved patient recovery, and it could be considered when femoral access is not available.

### Study limitations

We report single-center experience of alternative-access TAVR using the SAPIEN 3 device. Due to limited sample sizes, comparisons among the groups could be underpowered, and therefore, some of the results were suggestive but not conclusive. These groups also differ in some baseline features, and the small patient numbers limited our ability to perform propensity-score matching. Future large studies could help to elucidate this matter. Due to our early experience, long-term outcomes of the study cohorts were lacking.

## Conclusion

TAx access for TAVR with the SAPIEN3 valve is a safe alternative to TF-TAVR. Despite the patients undergoing TAx-TAVR procedures with higher risk profiles, we have shown that excellent procedural and short-term clinical outcomes can be achieved using our current approach to access route selection. TAx access offers advantages of improved recovery over TT access, and appears to be a superior alternative-access option for TAVR. It could be preferred when TF access is not feasible.

## Data Availability

Supporting data are available through the corresponding author on reasonable request.

## Abbreviations

AKI: Acute kidney injury
LOS: Length of stay
STS-PROM: Society of Thoracic Surgeons predicted risk of mortality
TA: Transapical
TAo: Transaortic
TAVR: Transcatheter aortic valve replacement
TAx: Transaxillary
TF: Transfemoral
TT: Transthoracic
VARC: Valve Academic Research Consortium
